# Understanding the pathophysiology of NOMID arthropathy for drug discovery by iPSCs technology

**DOI:** 10.1186/1546-0096-13-S1-P195

**Published:** 2015-09-28

**Authors:** K Nakagawa, Y Okuno, R Nishikomori, K Yokoyama, T Tanaka, T Kawai, T Yasumi, K Umeda, N Nakayama, J Toguchida, M Hagiwara, T Heike

**Affiliations:** 1 Kyoto University Graduate School of Medicine, Department of Pediatrics, Kyoto, Japan; 2 Kyoto University Graduate School of Medicine, Department of Anatomy and Developmental Biology, Kyoto, Japan; 3 The University of Texas Health Science Center at Houston Medical School, Institute of Molecular Medicine, Houston, TX, USA; 4 Center for iPS Cell Research and Application, Kyoto University, Department of Cell Growth and Differentiation, Kyoto, Japan

## Introduction and objectives

NOMID, also known as CINCA syndrome, is a dominantly inherited autoinflammatory disease caused by *NLRP3* mutations. The pathophysiology of NOMID is explained by gain of function mutation of NLRP3, which activates NLRP3 inflammasome and produce an excess of IL-1β. This mechanism is supported by clinical observation that anti-IL-1 therapy is effective on its systemic inflammation. However, one of its characteristic features, epiphyseal overgrowth, is considered to be resistant to anti-IL-1 therapy, which raises a question that other mechanism than NLRP3 inflammasome may play a role in the epiphyseal overgrowth. In this study, we investigated the effect of mutated NLRP3 on chondrocytes using induced pluripotent stem cells (iPSCs) derived from NOMID patients, and tried to identify drugs to treat the abnormal chondrocytes overgrowth.

## Methods

We established isogenic iPSCs with wild-type or mutant NLRP3 from 2 NOMID patients with NLRP3 somatic mosaicism. We differentiated the iPSCs into chondrocytes, and the phenotypes of chondrocytes derived from iPSCs with wild-type NLRP3 and mutant ones were compared, particularly the size of the chondrocyte tissue produced.

## Results

Mutant iPSCs produced larger chondrocyte masses than wild-type iPSCs owing to glycosaminoglycan overproduction. We also observed increased expression of SOX9, which is a chondrocyte master-regulator, on chondrocyte masses derived from mutant iPSCs. In addition, in vivo transplantation of mutant cartilaginous pellets into immunodeficient mice NOG caused disorganized endochondral ossification. Enhanced chondrogenesis observed in chondrocyte masses derived from mutant iPSCs was independent of caspase-1 and IL-1, and thus probably the NLRP3 inflammasome. Reporter assays using the human *SOX9* promoter in chondroprogenitor cells revealed that the proximal CREB/ATF-binding site was critical for SOX9 overexpression caused by mutated NLRP3. These data was correlated with increased levels of cAMP and phosphorylated CREB in mutant chondroprogenitor cells. We are now developing high throughput screening system to identify compounds to inhibit the abnormal chondrocytes overgrowth.

## Conclusion

Our findings indicate that the intrinsic hyperplastic capacity of NOMID chondrocytes is dependent on the cAMP/PKA/CREB pathway, independent of the NLRP3 inflammasome.

